# Conservation Measures and Future Perspectives for Europe’s Most Threatened Frog: The Action Plan for Karpathos Water Frog (*Pelophylax cerigensis*)

**DOI:** 10.3390/biology15030273

**Published:** 2026-02-03

**Authors:** Apostolos Christopoulos, Vassia Spaneli, Dino Protopappas, Panayiotis Pafilis

**Affiliations:** 1 Section of Zoology and Marine Biology, Department of Biology, National and Kapodistrian University of Athens, Panepistimioupolis, GR-15772 Athens, Greece; 2 Hellenic Herpetological Society, Knossos Avenue, GR-71409 Heraklion, Greece; vassiaspan@yahoo.gr; 3 Management Unit South-Eastern Aegean Protected Areas, Natural Environment and Climate Change Agency, GR-11525 Athens, Greece; alimouda2000@yahoo.com; 4 Museum of Zoology, National and Kapodistrian University of Athens, Panepistimioupolis, GR-15772 Athens, Greece

**Keywords:** Aegean Archipelago, anuran amphibians, ecological observations, endemic species, Greece, Karpathos Island, water scarcity

## Abstract

Until recently, the Karpathos water frog (*Pelophylax cerigensis*) was considered endemic to Karpathos Island (Greece) and has recently been reclassified by the IUCN as Endangered (EN), having been previously assessed as Critically Endangered (CR). The species faces severe threats primarily associated with the scarcity of freshwater bodies in the southern Aegean Sea. Over the past decade, demographic assessments have revealed a marked population decline, driven by the intensifying effects of climate change, including reduced rainfall, and increasing summer temperatures. In addition, the few natural ponds that persist during the dry summer months are often shared with the Levantine freshwater crab (*Potamon potamios*), resulting in increased frog mortality due to predation. Despite these challenges, recent developments provide cautious optimism. These include the construction of a dam in southern Karpathos and the taxonomic reassessment of the water frog population on the neighboring island of Rhodes as conspecific with *P. cerigensis*. In response to the species’ precarious status, the Hellenic Herpetological Society designed and implemented a National Action Plan aimed at the protection and conservation of the Karpathos water frog. The Action Plan includes a series of targeted mitigation measures, such as the construction of artificial ponds to retain water during the summer, as well as a hydrological study addressing the seasonal drying of the ecologically important Eleimonitria spring. A key component of the Action Plan involves education and outreach initiatives targeting primary school students, local residents, and visitors, highlighting the frog’s ecological importance and conservation needs. Informational brochures will be distributed across the island to raise awareness of the species’ conservation status and the importance of safeguarding its habitat. The implementation of this Action Plan aims to secure the long-term survival of the Karpathos water frog and to strengthen integrated conservation efforts across its extremely limited range.

## Simple Summary

The Karpathos water frog (*Pelophylax cerigensis*) was until recently known to occur only on the island of Karpathos, Greece, and is classified as Endangered (EN) by the IUCN. Its survival is seriously threatened by the lack of freshwater habitats in the southern Aegean, a problem that has worsened due to reduced rainfall and higher summer temperatures. Population studies over the last decade show a strong decline in numbers. During the dry summer months, the remaining natural ponds are often shared with the Levantine freshwater crab, which increases frog mortality through predation. Despite these challenges, some positive developments have occurred, including the construction of a dam in southern Karpathos and the recognition of the Rhodes water frog population as the same species. To address the species’ critical status, the Hellenic Herpetological Society developed a National Action Plan. This plan includes creating artificial ponds, conducting hydrological studies, and implementing education and awareness programs to support the frog’s long-term conservation.

## 1. Introduction

Amphibian survival is strongly dependent on high levels of environmental humidity [1,2,3]. This physiological dependence is reflected in the disproportionately high number of amphibian species threatened with extinction worldwide, which currently reaches approximately 41% according to the IUCN [4]. Among the primary drivers of this decline is the increasing scarcity of freshwater bodies, a process that has been exacerbated by global climate warming [5,6]. In addition to climate-related pressures, amphibian populations are affected by a suite of anthropogenic and natural stressors, including pollution [7,8,9], infectious diseases [10,11,12], habitat degradation and fragmentation [13,14,15], invasive species [16,17], and road mortality [18,19,20]. Collectively, these pressures have rendered amphibians one of the most vulnerable vertebrate groups globally.

Climate warming represents one of the most severe environmental challenges of the 21st century [21]. Rising global temperatures are disrupting ecosystems worldwide, with profound consequences for biodiversity and ecosystem functioning [22,23]. Beyond direct physiological effects on individual species, climate change alters ecological interactions and destabilizes community dynamics, leading to cascading impacts across trophic levels [24]. Furthermore, climate warming increases the frequency and intensity of extreme weather events, such as droughts and heatwaves, which impose additional stress on already vulnerable species and habitats [25].

Amphibians are particularly sensitive to climate change due to their biphasic life cycle and dependence on both aquatic and terrestrial environments [1,26,27]. Rising temperatures can disrupt breeding phenology, as many amphibian species require narrow thermal windows for successful reproduction [28,29]. Altered precipitation regimes, including prolonged droughts, can lead to the desiccation of breeding sites such as ponds, springs, and temporary water bodies [30]. Climate change may also facilitate the spread of pathogens, such as chytrid fungi, while simultaneously reducing immune competence in amphibians [31,32]. In addition, changes in temperature and water availability influence dispersal capacity, prey availability, and predator–prey interactions, ultimately reshaping amphibian communities [1]. The combined effects of these processes can drive local population declines and, in extreme cases, extinction. Consequently, habitat conservation and targeted mitigation measures aimed at buffering the impacts of climate change are urgently required [33].

In response to the global amphibian crisis, species-specific action plans and conservation programs have been developed to safeguard threatened taxa and their habitats [34,35,36,37,38]. Such initiatives typically combine population monitoring, habitat restoration and management, environmental assessment, and public awareness and engagement [39].

The Karpathos water frog (*Pelophylax cerigensis* Beerli, Hotz, Tunner, Heppich & Uzzell, 1994) is widely regarded as Europe’s most endangered anuran amphibian, with its total population estimated at approximately 500–600 individuals [40]. The species was considered endemic to Karpathos Island, where freshwater habitats are extremely scarce, but it has recently been confirmed on Rhodes, another island in the southern Aegean Sea (Greece). One of the main pressures affecting the species is the pronounced reduction in rainfall over the past three decades [41,42], which has drastically limited the availability of suitable aquatic habitats. As a result, only a small number of ponds and rivulets persist during the prolonged dry period on Karpathos, which typically extends from late March to early November [40,43].

To address the rapid decline of *P. cerigensis*, the National and Kapodistrian University of Athens, in collaboration with WWF Greece, developed a National Action Plan for the species. The plan was implemented by the Hellenic Herpetological Society between 2020 and 2026. In the present study, we outline the key components of this Action Plan, describe the challenges encountered during its implementation, present the results achieved to date, and discuss the progress made towards securing the long-term conservation of the Karpathos water frog.

## 2. Materials and Methods

### 2.1. Study System

Karpathos Island is the second largest island of the Dodecanese complex, with a total area of 324.7 km^2^ and a maximum elevation of 1215 m at Kali Limni Mountain [44]. Karpathos began to form geologically about 12 million years ago with the opening of the Mid-Aegean Trench [45]. It was initially isolated around 8 million years ago, while its final separation from Rhodes occurred approximately 3.5 million years ago [46,47]. This long-term isolation has resulted in high levels of biodiversity, including numerous endemic taxa and largely intact natural landscapes [48].

The climate of Karpathos is typically Mediterranean, characterized by hot, dry summers and mild, relatively humid winters. However, the northern part of the island experiences distinct microclimatic conditions, with increased exposure to strong winds and generally drier and cooler conditions compared to the rest of the island [49]. Due to its ecological importance, a substantial proportion of Karpathos is included in the Natura 2000 network, with two designated protected areas (GR4210002 and GR4210003).

**Figure 1:**
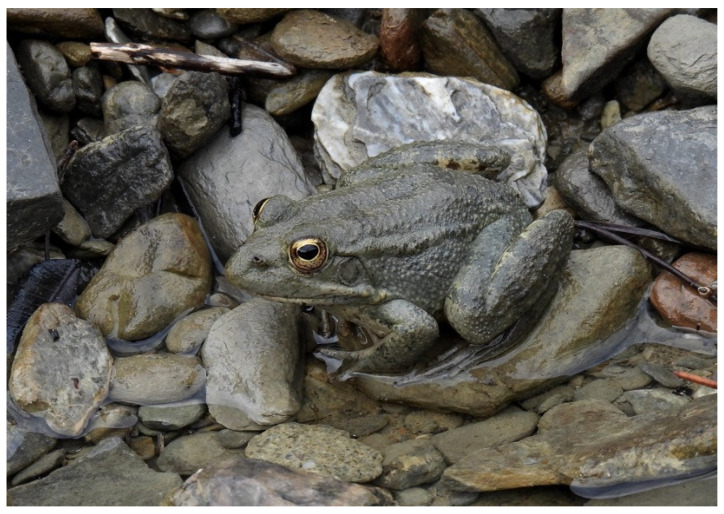
An adult Karpathos water frog in the Argoni area (Photo by A.C.).

The Karpathos water frog (*Pelophylax cerigensis*) is a medium-sized anuran, with a snout–vent length (SVL) ranging from approximately 5 to 7 cm. Dorsal coloration varies from brown and brown-grey to olive-brown, typically with numerous small dark spots and blotches (Figure 1). In some individuals, a light yellow-green or cream-colored vertebral stripe is present [41,50]. The species is primarily diurnal and remains active throughout the year, although during the summer months, activity shifts towards dusk. Breeding takes place in spring [41]. Feeding is opportunistic and largely dependent on prey availability, with Coleoptera, Hymenoptera, and spiders constituting the main prey groups [43].

Until recently, *P. cerigensis* was considered endemic exclusively to Karpathos Island. However, recent phylogenetic and molecular evidence has demonstrated that the water frog population on the neighboring island of Rhodes also belongs to this species [51,52]. The Karpathos water frog, which until recently was classified as Critically Endangered (CR), is now listed as Endangered (EN) on both the IUCN Red List [53] and the Greek Red List [54].

### 2.2. Study Area

**Figure 2:**
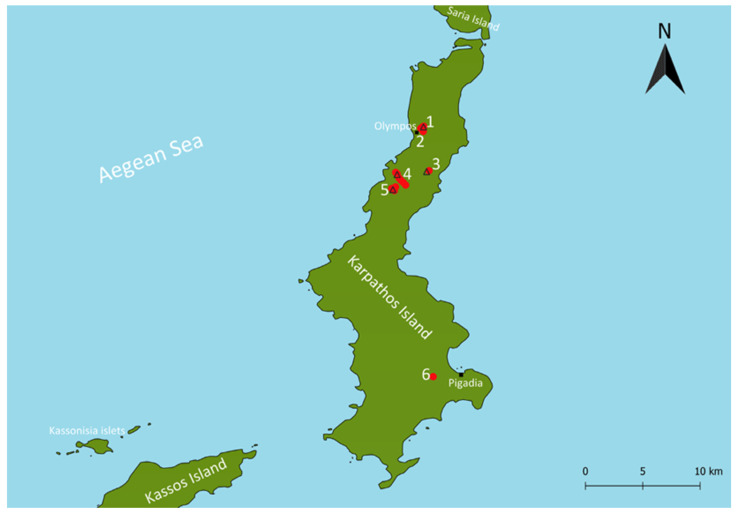
Map of Karpathos Island showing the distribution areas of the Karpathos water frog (red dots); (1) Panagia Eleimonitria Spring, (2) Olympos stream system, (3) Nati rivulet, (4) Argoni rivulet, (5) Achamantia area, and (6) Schina Dam. Triangles indicate the locations where new ponds were constructed to enhance the species’ habitat.

The Action Plan primarily focused on the northern part of Karpathos Island, where the main extant populations of the species occur. The principal distribution areas include the rivulets of Olympos and Achamantia, as well as the Argoni and Nati areas (Figure 2). These freshwater systems constitute the core habitats supporting the remaining populations of *P. cerigensis* on the island.

Vegetation surrounding the rivulets is dominated by phrygana and sparse shrub formations. Characteristic plant species include thyme (*Thymbra capitata*), thorny burnet (*Sarcopoterium spinosum*), lentisk (*Pistacia lentiscus*), and, in some areas, open stands of eastern Mediterranean pine (*Pinus brutia*). In several locations along the rivulets, thermo-Mediterranean riparian galleries are present, with dominant vegetation consisting of oleander (*Nerium oleander*) and spiny rush (*Juncus articulatus*) [43].

In addition to natural water bodies, small frog populations were also recorded in traditional artificial structures, such as concrete water tanks and livestock watering troughs, which locally function as alternative refugia during dry periods.

### 2.3. Actions and Methods

The overarching aim of the Action Plan was to secure the long-term survival of the Karpathos water frog and to promote integrated conservation efforts across its limited range. To achieve this goal, a set of priority actions and mitigation measures was identified and implemented, structured around four main objectives.

#### 2.3.1. Halting and Reversing Population Decline

To mitigate the effects of prolonged drought and habitat loss, new artificial ponds were constructed to retain water during dry periods and function as refugia for the species. In parallel, a hydrogeological study was conducted at the Panagia Eleimonitria Spring, followed by targeted technical interventions aimed at restoring water availability. The reactivation of this spring had a positive effect on the frog population inhabiting the Olympos rivulets.

#### 2.3.2. Improvement of Habitat Quality

Efforts were made to enhance the capacity of existing habitats to support viable frog populations. This included the regular maintenance of natural and artificial water bodies, as well as the removal of waste, debris, and sediment from all known sites occupied by the species.

#### 2.3.3. Enhancement of Biological and Ecological Knowledge

**Figure 3:**
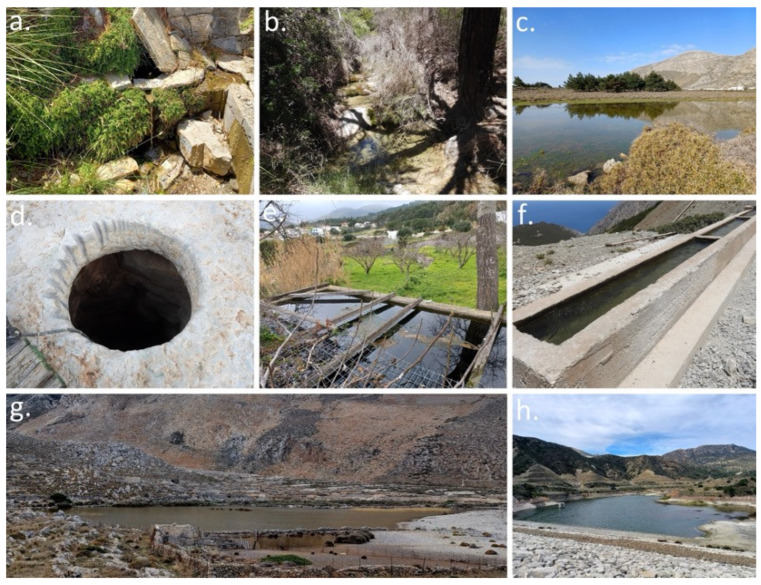
(**a**) The Ligo Nero Spring located on the east side of central Karpathos. (**b**) The rivulet Forokli at Panagia Prastiotissa area. (**c**) Temporal pond in Lastos Plateau. (**d**) Well in the Achordaea area. (**e**) Concrete water tank in the settlement of Stes. (**f**) Livestock watering trough in northern Karpathos. (**g**) The marsh of Tristomo area. (**h**) Schina Dam at the area of Pigadia (Photos by A.C.).

A key priority was the investigation of the taxonomic status and genetic structure of the Rhodes Island populations, following earlier suggestions that they might belong to the same species as the Karpathos population. Molecular analyses were conducted, and genetic diversity was assessed at the population level [51,55,56]. To assess the full extent of the species’ distribution on Karpathos Island, extensive field surveys were carried out across a large number of water bodies. A total of 219 sites were surveyed, including concrete water tanks (*n* = 152), springs (*n* = 27), streams and rivulets (*n* = 15), livestock watering troughs (*n* = 8), cisterns (*n* = 7), temporary ponds (*n* = 5), wells (*n* = 3), a marsh (*n* = 1), and a dam (*n* = 1) (Figure 3a–h). In addition, frog populations were systematically monitored throughout the duration of the project, with surveys conducted twice annually using line transects and point counts. Further emphasis was placed on documenting the species’ ecology through systematic field observations.

#### 2.3.4. Public Awareness and Stakeholder Engagement

To foster local support for conservation actions, a targeted educational program was developed for primary school students on Karpathos. In addition, an informational brochure, available in both Greek and English, was produced to communicate the ecological importance of the species and the conservation measures implemented. The brochure was distributed to local residents and visitors to the island.

## 3. Results

### 3.1. Pond Construction

**Figure 4:**
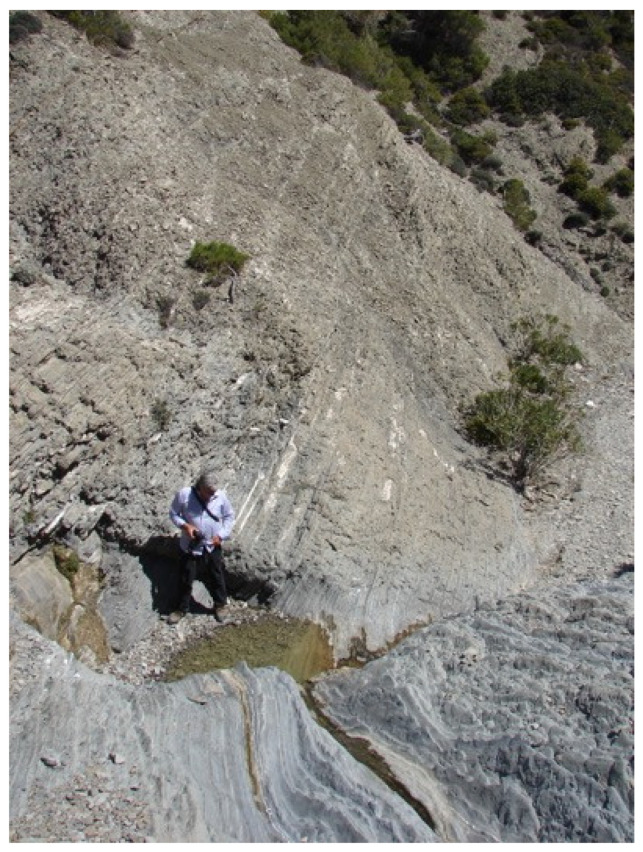
Water scarcity in the Nati rivulet during spring (Photo by P.P.).

Small artificial ponds were constructed as part of the Action Plan to mitigate the effects of summer drought and the drying of natural water bodies. Their construction was approved by the local Management Unit of Protected Areas of the Natural Environment and Climate Change Agency (N.E.C.C.A.) and licensed by the Dodecanese Department of Forest Administration (Approval No. A.Π. 58826). Summers on Karpathos are characterized by prolonged aridity, during which many rivulets inhabited by the species dry out completely (Figure 4). The newly constructed ponds were therefore designed to provide permanent or semi-permanent refugia for frogs during dry periods.

**Figure 5:**
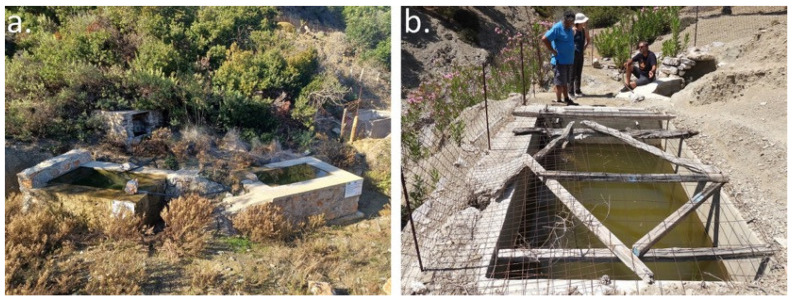
(**a**) Two of the newly built water tanks in the area of Achamantia. (**b**) An older water tank connected with the new water tanks in the area of Argoni (Photos by A.C.).

In total, five water tanks were constructed following similar design specifications: approximately 2 m in length, 1.5 m in width, and 1 m in depth, with walls approximately 40 cm thick. Site selection was based on three main criteria: accessibility for construction machinery, availability of adequate space, and proximity to a spring capable of supplying water to the tanks. The ponds were built using local stone to ensure visual and ecological compatibility with the surrounding landscape, while the interior surfaces were coated with cement mortar to ensure waterproofing. Small ramps were incorporated both inside and outside the tanks to facilitate frog movement in and out of the water. In locations adjacent to slopes, low retaining walls (30–50 cm high) were constructed to prevent sediment and eroded material from entering the tanks. Water was supplied to the tanks via polyvinyl chloride (PVC) hoses connected to nearby springs (Figure 5a,b).

### 3.2. Panagia Eleimonitria Spring Study

The Panagia Eleimonitria Spring, located in the Olympos area, historically supported a section of a rivulet inhabited by a small frog population. In recent years, however, water discharge from the spring has declined substantially and, during certain visits both before and during the implementation of the Action Plan, it ceased entirely, primarily in the summer and autumn months. During the early years of the project, both adult frogs and tadpoles were recorded at the spring, whereas following periods of water depletion, only few adult individuals, or none at all, were observed (see Table 1), indicating a marked reduction in local frog presence. To address this issue, a hydrological study was commissioned to investigate the spring’s hydrogeological characteristics and identify potential measures to secure a more stable water supply. The study found that the reduced yield of the Eleimonitria Spring is mainly due to natural hydrogeological factors, including a 33% decrease in rainfall over the past 20–25 years, particularly summer droughts, and the local geology, while human activities mainly affect the movement of water after it leaves the spring toward the rivulet [42].

Based on the findings of this study, mild technical interventions were implemented. These included the construction of an artificial pond fed by an existing upstream tank supplied by the spring. Overflow from both the new and the pre-existing tanks was directed into the rivulet, thereby ensuring a more continuous flow of water. As the summer refugia previously used by frogs in this rivulet were drying out completely, the intervention is expected to maintain water availability throughout the year, provided that the spring continues to discharge. The construction was approved by the Directorate for the Management of Protected Areas (Sector B) of N.E.C.C.A. and licensed by the Dodecanese Department of Forest Administration (Approval No. A.Π. 212954).

### 3.3. Maintenance of Existing Water Bodies

At the onset of the Action Plan, surveys were conducted at all known frog habitats in northern Karpathos, including both natural and artificial water bodies, in order to assess their condition. Overall, most rivulets and water tanks were found to be in good condition, with minimal evidence of human disturbance, littering, or infilling by debris. In a limited number of cases, however, water tanks contained accumulated debris or small amounts of discarded waste were observed within rivulets, specifically in a few rivulet puddles of the Olympus stream system located near a residential area, while the more remote streams showed no signs of littering or infilling.

All foreign materials were removed from affected sites to prevent further degradation and reduce the risk of complete infilling by soil and stones. These maintenance actions contributed to preserving the functionality of existing water bodies as suitable frog habitats.

### 3.4. Molecular Analyses

Molecular analyses were conducted on samples collected from Karpathos and Rhodes during the first year of the project. The results confirmed that frog populations from both islands belong to the same species, forming a well-supported monophyletic clade with low genetic divergence between populations [52].

### 3.5. Surveys of Water Bodies on Karpathos

Most of the surveyed areas across the island did not reveal new frog populations. However, a small population was newly discovered during this project at Schina Dam, near the island’s capital, Pigadia, in southern Karpathos. The dam, completed in 2020–2021, covers an area of approximately 5.42 ha. The presence of frogs at this site is presumed to be the result of accidental human-mediated translocation.

### 3.6. General Ecological Observations

**Figure 6:**
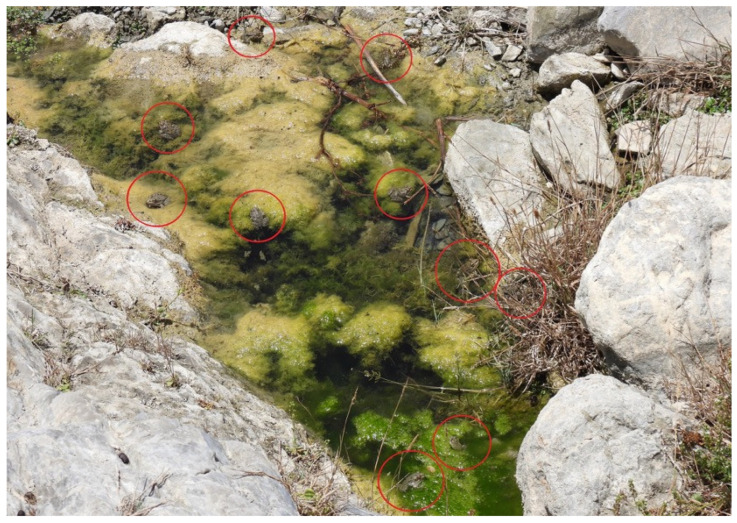
Seasonal frog aggregation in a remaining puddle of a rivulet in the Olympos area during summer drought, when most of the streambed dries out. Red circles indicate the position of individual frogs (Photo by A.C.).

Field surveys revealed that frogs actively migrate over short distances within rivulets and streams, typically several tens of meters, among nearby puddles in response to declining water availability. These movements were occasional and involved individual frogs rather than large groups. As shallow pools dry out, individuals move upstream to sections where small puddles retain water, even during the driest periods of the year. Consequently, large aggregations of frogs (up to 37 individuals) were observed within single puddles during summer and autumn, irrespective of puddle size (Figure 6). In contrast, during winter and spring, when water availability increases, frogs were more evenly distributed across multiple pools, typically with 1–5 individuals per puddle depending on size.

While adult frogs are capable of relocating between water bodies, tadpoles are highly vulnerable to desiccation. On multiple occasions, large numbers of tadpoles that had not yet completed metamorphosis were found dead in puddles that had dried out completely during July and September.

**Figure 7:**
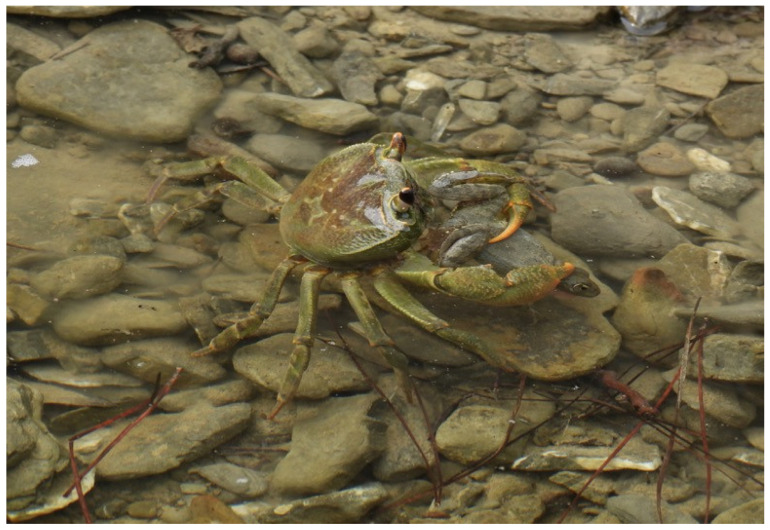
An incident of predation of a Karpathos water frog by the Karpathos freshwater crab in shallow waters in Argoni rivulet during drought conditions (Photo by A.C.).

Predation pressure increased markedly in the few remaining water bodies during drought conditions. The most frequently observed predator was the Karpathos freshwater crab (*Potamon karpathos*) (Figure 7). Although the two species have coexisted historically, the scarcity of suitable habitats forces both to occupy the same limited water bodies for extended periods, leading to repeated predation events [57]. In addition, six species of migratory batrachophagous birds (*Ardea cinerea*, *Ardea purpurea*, *Egretta garzetta*, *Ardeola ralloides*, *Nycticorax nycticorax*, and *Circus aeruginosus*) were observed feeding in habitats occupied by the frog. Direct predation on tadpoles or juveniles was confirmed for *E. garzetta*, *A. ralloides*, and *N. nycticorax*. A single record of frog remains in beech marten (*Martes foina*) droppings suggests occasional predation by mammals, although further study is required to assess its significance.

### 3.7. Population Monitoring and Colonization of Newly Constructed Ponds

Continuous population monitoring was conducted across all study sites to evaluate demographic structure, temporal trends, and site-specific population dynamics. The number of frogs recorded was systematically documented each year, with individuals categorized as adults, juveniles, and tadpoles, providing insights into overall population fluctuations and long-term trends. These data allowed for an assessment of population health and the identification of sites with higher or lower densities, contributing to a broader understanding of species status on the island. The results of population monitoring are presented in Table 1.

One of the central objectives of the Action Plan was the creation of artificial ponds to expand available habitat. The newly constructed ponds were systematically monitored to assess their use by the species. Results were encouraging; despite their very recent construction, field observations indicated early colonization by the target species, with adult frogs present and breeding observed in Argoni ponds during the first year of their construction, and a small number of tadpoles recorded in Achamantia ponds during the second year, indicating successful use of these artificial habitats for both refuge and reproduction. These observations should be regarded as indicative rather than conclusive, and a systematic, long-term monitoring scheme is required to reliably assess the effectiveness of the constructed ponds in supporting amphibian populations under prolonged drought conditions. In Table 2, yearly counts of individuals have been included to provide a more detailed picture of colonization success.

### 3.8. Education and Awareness

A six-lesson educational package was developed specifically for primary school students. Each lesson targeted defined learning outcomes and combined classroom-based activities with field exercises, allowing students to directly observe the frog and its habitat. Through these activities, students explored local environmental conditions, temporal and spatial changes, and the ecological role of the species, fostering awareness of the relationship between biodiversity and human well-being.

In addition, two outreach actions targeted the wider public. Presentations on the biology and conservation of the Karpathos water frog were delivered in all primary schools on the island. Furthermore, an informational brochure, produced in both Greek and English, was published and will be distributed to residents and visitors, highlighting the species’ conservation status and the measures implemented under the Action Plan.

## 4. Discussion

The conservation actions implemented for the Karpathos water frog (*P. cerigensis*) represent a targeted response to the severe and ongoing pressures faced by one of Europe’s most threatened amphibian species. The results of the Action Plan demonstrate that carefully designed habitat-based interventions, combined with systematic monitoring and public engagement, can mitigate some of the immediate effects of drought and habitat loss in insular environments characterized by extreme water scarcity.

The construction of artificial ponds proved to be one of the most effective measures implemented under the Action Plan. Prolonged summer droughts on Karpathos frequently result in the complete desiccation of natural rivulets and puddles, leading to high mortality rates, particularly among tadpoles that are unable to relocate. The rapid colonization of newly constructed ponds, including the presence of both adult frogs and tadpoles during the year of construction and the subsequent year, indicates that these artificial water bodies function successfully as refugia and breeding sites. Similar findings have been reported in other amphibian conservation and monitoring programs, where artificial or restored aquatic habitats have partially compensated for the loss of natural water bodies under changing climatic conditions [30,58,59,60]. In the context of Karpathos, where freshwater availability is extremely limited, such interventions are likely to play a critical role in sustaining local populations, particularly in areas such as Achamantia, where population size is currently very low.

The hydrological interventions at the Panagia Eleimonitria Spring further highlight the importance of maintaining year-round water availability in key breeding and refuge sites. The decline in water discharge from the spring had previously resulted in the near-collapse of the local frog population. The redirection and stabilization of water flow through mild technical measures are expected to improve habitat stability and reduce seasonal bottlenecks. Although the long-term effectiveness of this intervention will depend on future precipitation patterns and the persistence of spring discharge, the approach demonstrates how site-specific hydrological management can contribute meaningfully to amphibian conservation in water-limited landscapes.

Field observations revealed pronounced seasonal shifts in frog distribution, driven primarily by water availability. During dry periods, frogs congregated in a small number of remaining puddles, often at high densities, whereas in wetter seasons, individuals were more evenly distributed across available habitats. This behavioral response increases vulnerability to predation and density-dependent mortality during droughts. The repeated observation of predation by the Karpathos freshwater crab (*P. karpathos*), as well as by migratory birds, underscores the indirect effects of habitat contraction. While predation is a natural ecological process, the forced co-occurrence of predators and prey in a limited number of shrinking water bodies likely amplifies its impact beyond natural levels. Similar interactions have been documented in other drought-affected amphibian systems, where reduced habitat availability intensifies predator–prey encounters and accelerates population decline [1,61,62].

Tadpole mortality emerged as a critical bottleneck for population persistence. The repeated loss of entire cohorts due to puddle desiccation highlights the vulnerability of early life stages to climatic extremes. This pattern is consistent with global evidence indicating that larval mortality driven by drought is a major contributor to amphibian population declines, particularly in species with restricted distributions and limited dispersal capacity [30,58]. The presence of tadpoles in newly constructed ponds suggests that artificial water bodies may partially alleviate this bottleneck by extending hydroperiods during the breeding season.

The molecular confirmation that frog populations on Karpathos and Rhodes belong to the same species has important conservation implications. The species’ persistence is supported by the presence of geographically isolated populations, which reduces the risk of total extinction if one population is lost, and emphasizes the importance of island-specific management strategies. Each population is exposed to distinct environmental pressures, and conservation actions should aim to preserve local genetic and ecological characteristics rather than treating the species as a single homogeneous unit.

The unexpected discovery of a small population at the Schina Dam highlights both the species’ capacity to exploit newly available aquatic habitats and the potential role of human-mediated translocation. Although the long-term viability of this population remains uncertain, large artificial reservoirs may offer relatively stable aquatic environments in otherwise arid landscapes. Nevertheless, reliance on such habitats should be approached cautiously, as they may introduce new threats, including altered predator communities and water management practices beyond conservation control.

Beyond direct habitat interventions, education and public awareness emerged as essential components of the Action Plan. Engaging local communities, particularly younger generations, fosters long-term stewardship and reduces the likelihood of habitat degradation. The generally high environmental awareness reported among residents of northern Karpathos suggests that conservation initiatives can build upon existing positive attitudes, reinforcing local involvement in species protection.

Overall, the results of this study indicate that while the Karpathos water frog remains highly vulnerable to climate change, drought, and habitat limitation, targeted and adaptive management actions can slow or partially reverse local declines. Continued long-term monitoring will be essential to evaluate population trends, assess the durability of implemented measures, and refine conservation strategies in response to ongoing environmental change. The Action Plan for *Pelophylax cerigensis* provides a valuable framework that may be applicable to other insular amphibian species facing similar challenges under increasingly arid climatic conditions.

## 5. Conclusions

Amphibians are the most threatened vertebrate group globally, and the Karpathos water frog (*Pelophylax cerigensis*) exemplifies the vulnerability of insular species exposed to increasing climatic stress and habitat limitation. The implementation of the National Action Plan has produced encouraging outcomes, demonstrating that targeted, science-based interventions can mitigate key threats acting on critically endangered amphibians.

Habitat restoration through artificial pond construction, hydrological management of critical springs, systematic population monitoring, and genetic assessment have collectively improved habitat availability and supported population persistence. The rapid use of newly created water bodies by adults and tadpoles highlights their effectiveness in buffering drought impacts and reducing larval mortality. Confirmation that the Rhodes and Karpathos populations belong to the same species further refines the conservation framework for *P. cerigensis*.

Nevertheless, significant challenges remain. Climate change-driven reductions in rainfall, rising temperatures, and increased predation pressure in shrinking habitats continue to threaten long-term viability. Sustained maintenance and expansion of artificial and semi-natural refugia, combined with adaptive management, will be essential in this water-limited island system.

Community engagement and education are equally crucial. Raising awareness among residents and visitors fosters local stewardship and strengthens long-term conservation success. In conclusion, although full recovery remains uncertain, the actions implemented to date provide a strong foundation for the long-term conservation of the Karpathos water frog. This Action Plan offers a valuable model for the conservation of insular amphibians facing similar challenges under ongoing environmental change.

## Figures and Tables

**Table 1:** Population monitoring of Karpathos water frog across the island (2021–2025). Annual counts of adults, juveniles, and tadpoles across all known distribution areas, providing demographic data and site-specific population dynamics. The Nati rivulet is not included, as the species has not been recorded there since 2020.

Location	Year	Adults	Youngs	Tadpoles
Argoni Rivulet	2021	72	18	338
	2022	75	60	345
	2023	94	11	173
	2024	75	7	220
	2025	90	27	318
Eleimonitria Spring	2021	2	0	10
	2022	1	0	0
	2023	0	2	0
	2024	0	0	0
	2025	0	0	0
Olympos Rivulet, N–NNE	2021	54	10	34
	2022	15	3	10
	2023	16	9	32
	2024	14	1	70
	2025	21	6	75
Olympos Rivulet, E	2021	22	0	0
	2022	30	7	15
	2023	50	0	10
	2024	17	4	45
	2025	24	14	35
Achamantia area	2021	1	0	0
	2022	7	0	0
	2023	1	0	0
	2024	3	0	10
	2025	4	5	1
Schina Dam (Pigadia)	2021	0	0	0
	2022	0	0	0
	2023	0	0	0
	2024	15	0	0
	2025	2	0	0

**Table 2:** Colonization of newly constructed ponds by Karpathos water frog (2023–2025). Yearly records of adults, juveniles, and tadpoles in artificial ponds in the Argoni and Achamantia areas, providing demographic data and indicating early colonization and reproductive activity. The newly constructed ponds in Nati and Eleimonitria are not included, as no frogs were recorded there during monitoring.

Location	Year	Adults	Youngs	Tadpoles
Argoni pond 1	2023	1	0	0
	2024	2	0	2
	2025	2	0	0
Argoni pond 2	2023	2	0	4
	2024	6	1	7
	2025	1	2	2
Achamantia pond 1	2023	0	0	0
	2024	0	0	1
	2025	0	0	0
Achamantia pond 2	2023	0	0	0
	2024	0	0	10
	2025	0	1	1

## Data Availability

The raw data supporting the conclusions of this article will be made available by the authors on request.
